# Relationship of Milk Odd- and Branched-Chain Fatty Acids with Urine Parameters and Ruminal Microbial Protein Synthesis in Dairy Cows Fed Different Proportions of Maize Silage and Red Clover Silage

**DOI:** 10.3390/ani10020316

**Published:** 2020-02-18

**Authors:** Edwin Westreicher-Kristen, Joaquín Castro-Montoya, Mario Hasler, Andreas Susenbeth

**Affiliations:** 1 Institute of Animal Nutrition and Physiology, Christian-Albrechts-Universität zu Kiel, 24118 Kiel, Germany; 2 Schothorst Feed Research BV, P.O. Box 533, 8200 AM Lelystad, The Netherlands; 3 Department of Animal Nutrition and Rangeland Management in the Tropics and Subtropics, Institute of Agricultural Sciences in the Tropics, University of Hohenheim, 70599 Stuttgart, Germany; 4 Lehrfach Variationsstatistik, Christian-Albrechts-Universität zu Kiel, 24118 Kiel, Germany

**Keywords:** purine derivative, microbial protein, odd- and branched-chain fatty acids, red clover

## Abstract

**Simple Summary:**

The purpose of this study is to assess the relationship of milk odd- and branched-chain fatty acids (OBCFA) with urinary purine derivates (PD) and estimated ruminal microbial crude protein (MCP). The correlations and regressions demonstrate that yields and concentrations of individual or total OBCFA are weakly related to urinary PD and are low to moderate markers of MCP synthesis. Nevertheless, milk OBCFA can still be seen as a promising method for predicting rumen function and microbial protein supply to the duodenum in dairy cows because MCP flow was not directly measured in this study but instead indirectly estimated probably comprising considerable deviations of the assumed values from the true ones.

**Abstract:**

The aim of this study was to evaluate the relationship of milk odd- and branched-chain fatty acids (OBCFA) with urinary purine derivates and estimated ruminal microbial crude protein (MCP) synthesis. Forty-four lactating Holstein cows were used in a 4 × 4 Latin square design with 21-day periods comprised of a 13-day adaptation phase to diet followed by an 8-day sampling phase. Differences in estimated MCP yield and milk OBCFA composition were found by feeding total mixed rations containing forage (maize silage, MS; red clover silage, RCS) and concentrates (0.75:0.25) with targeted proportions of RCS to MS of 0.15:0.60, 0.30:0.45, 0.45:0.30, and 0.60:0.15 on a dry matter basis. The MCP was estimated from the total urinary purine derivate (PD) excretion (MCPPD) and intakes of metabolizable energy (MCPME) or digestible organic matter (MCPdOM). The Pearson correlations of individual OBCFA with urinary parameters (uric acid, allantoin, PD and nitrogen) were generally weak (*r* = −0.37 to 0.55). Yields of individual OBCFA correlated positively with MCPME and MCPdOM (*r* = 0.21 to 0.55). The prediction of urinary PD concentration was moderate (R^2^ = 0.64) when including the proportion of iso-C17:0. The prediction of total PD excretion was low (R^2^ = 0.21) with yields of iso-C15:0, anteiso-C17:0, and iso-C16:0. The prediction of MCPPD was high (R^2^ = 0.99) when including the iso-C16:0 and cis-9 C17:1 concentrations, while those of MCPME and MCPdOM were low (R^2^ = 0.37 and 0.36, respectively) when including yields of iso-C15:0, cis-9 C17:1, and iso-C18:0. The correlations and regression analyses demonstrate that the estimated MCP synthesis and urinary PD excretion can be only moderately predicted by yields and concentrations of individual or total OBCFA in cow’s milk. However, milk OBCFA can still be seen as a promising, non-invasive method for predicting rumen function and microbial protein supply in dairy cows because MCP flow was not directly measured in this study but instead indirectly estimated probably comprising considerable deviations of the assumed values from the true ones.

## 1. Introduction

Microbial crude protein (MCP) synthesis in the rumen substantially contributes approximately 60 to 85% of the supply of amino acids to the duodenum [1]. Therefore, the estimation of MCP synthesis is an important prerequisite for formulating diets for dairy cows. However, quantifications of in vivo-MCP synthesis are limited and hampered due to the requirement of experiments with duodenal-cannulated animals. Therefore, as an alternative, the urinary excretion of purine derivates (PD) has been suggested as a non-invasive and reliable method to estimate the microbial N flow to the duodenum [2,3,4,5]. Nevertheless, the application of such a method still requires the collection of representative urine samples and the quantification of the total urine volume, which challenges its application in on-farm conditions. Therefore, there is interest to find reliable internal biomarkers in milk, as the latter can be sampled non-invasively and can be easily and routinely applied in practical dairy cattle feeding. Because odd- and branched-chain fatty acids (OBCFA) are synthesized de novo by ruminal microbes, incorporated into their cell membrane [6], and recovered to a great extent in the milk, the profiles of these fatty acids (FA) in milk have been also suggested as potential biomarkers for MCP flow to the duodenum. The content of OBCFA in milk has been related to differences in feeding strategies [7,8], the rumen fermentation pattern [6], to the duodenal flow of diaminopimelic acid and purine bases [9], and to ruminal bacterial populations [10]. Conversely, Dewhurst et al. [11] did not find any relationship between milk OBCFA and the duodenal flow of purine bases or to the duodenal flow of OBCFA. Additionally, Castro-Montoya et al. [12] found a weak relationship between milk OBCFA and PD in urine. In general, studies related to the use of OBCFA as a biomarker for MCP have shown contrasting results and indicate that more research is needed on this matter.

Red clover is an important forage legume for silage (RCS) production, characterized by a good crude protein (CP) content and high level of soluble CP in the form on non-protein nitrogen. Maize silage (MS) is an important forage and a significant source of soluble carbohydrates, which provides rapidly fermentable energy to ruminal microbes. Therefore, mixtures of RCS and MS are argued to have the potential to maximize the MCP in the rumen via better synchronization between the rapidly fermentable fractions of CPs and carbohydrates. To date, only few studies have evaluated mixtures of RCS and MS in terms of the performance of dairy cows [13,14,15,16], and no published study so far exits about the effect of RCS on OBCFA. Therefore, the main objectives of this study are to study the relationship of OBCFA with urinary PD and the estimated MCP synthesis in the rumen of dairy cows. Differences in the estimated MCP yield and milk OBCFA composition are found here by feeding different proportions of RCS and MS. We hypothesize (1) that the urinary PD and estimated flow of MCP to the duodenum is reflected by the content of OBCFA in the milk, and (2) that this positive relationship is close enough to be used as a reliable, easily available indicator for microbial synthesis in the rumen in both practical feeding and research.

## 2. Materials and Methods

### 2.1. Animals, Diets, and Experimental Design

The animal study reported herein was performed in accordance with the German Animal Welfare Act [17] and was approved by the Animal Welfare Commission of the Ministry of Energy, Agriculture, Environment, and Rural Areas of the Federal State of Schleswig-Holstein, Germany (V 242-72241.123-5). The data presented in this study were derived from a feeding trial described by Schulz et al. [16,18]. Succinctly, 44 German Holstein cows were used for the experiment. At the start of the trial, cows were randomly assigned to four groups according to milk yield, days in milk, lactation number, and body weight, averaging 38.7 ± 7.3 kg/d, 149 ± 103 d, 1.9 ± 1.1, and 623 ± 56 kg, respectively. Cows were kept in a free stall barn equipped with cubicles bedded with chopped straw and had free access to water. Dietary treatments were fed as total mixed rations (TMR) and were offered once a day at approximately 06:00 h for ad libitum consumption. The TMR was comprised of forage (MS and RCS) and a concentrate (0.75:0.25). The MS was replaced by RCS with targeted ratios of RCS in the TMR of 0.15 (RCS15), 0.30 (RCS30), 0.45 (RCS45), and 0.60 (RCS60) on a dry matter (DM) basis. The concentrates were based on lupine seeds, soybean meal (SBM), wheat, and a mineral and vitamin premix. The ingredients and chemical compositions of the four TMR are shown in Table 1, while the FA composition has already been reported in detail in Schulz et al. [18]. The methods used for the analyses of the chemical compositions of the four TMR are described in detail in Schulz et al. [16]. The feeding trial was based on a Latin square (4 × 4), with each of the four periods lasting 21 days. Each experimental period consisted of 13 days for diet adaption followed by 8 days for data and sample collection.

### 2.2. Sampling and Chemical Analyses

Cows were milked twice a day at 05:00 and 15:00 h and samples of milk were obtained from every cow once daily while milking on days 14 to 21 of each period, where sampling was alternated between the afternoon and morning milking periods on subsequent days. Afterwards, milk samples were stored at −20 °C. The analysis of FA in milk fat was run as a pooled sample per animal and period, as described by Schulz et al. [18]. Succinctly, the milk fat fraction was extracted according to the Roese–Gottlieb method [19]. Fatty acid methyl esters (FAME) were obtained from the extracted milk fat by transesterification [20]. Subsequently, the FA composition was determined by the gas chromatography procedure [21] as described by Castro-Montoya et al. [12]. Analyses of FAME were performed with an Agilent 7890A gas chromatograph equipped with a 60-m fused silica capillary column (i.d. 0.25 mm) coated with a 0.20-µm film of CP-Sil 88 (Agilent Technologies, Santa Clara, CA, USA), a split injection port (1:130), and a flame ionization detector. Hydrogen, flowing at a constant rate of 1.3 mL/min (94 kPa initial pressure), was the carrier gas. The temperature program was that as described by Molkentin and Giesemann [22]. Resulting chromatograms were evaluated with the Software EZChrom Elite 3.3.2 (Agilent Technologies). The reference milk fat CRM 164 (IRMM, Geel, Belgium) was used for the calibration of the major FA. Milk FAs were expressed as the proportion of total FAs (g/100 g of FA). Milk FAs reported in the current study are limited to those 10 FAs related to microbes leaving the rumen. In line with this, the FAs presented are odd-chain FAs (OCFA, C15:0, C17:0 and cis-9 C17:1), and branched-chain FAs (BCFA, iso-C14:0, iso-C15:0, iso-C16:0, iso-C17:0, iso-C18:0, anteiso-C15:0 and anteiso-C17:0). Yields of FAs (g/d) were calculated based on the FA proportion in total amount of FAs and yield of fat.

For the estimation of MCP, the concentrations of the urinary PD (uric acid and allantoin), nitrogen (N), and daily excretion values published by Schulz et al. [16] were used in the present study. For urine samples, the procedures for the collection and preparation of spot samples for the analysis of allantoin, uric acid, and N have been described in detail by Schulz et al. [16]. Succinctly, spot urine samples (~600 mL) were obtained from all cows once daily on days 14, 16, 18, and 20 at 07:30, 19:30, 15:30, and 11:30 h, respectively, by manual stimulation of the region around the vulva. This sampling design was applied to account for possible diurnal and day-to-day variations in the urinary excretion of PD and N [12]. Immediately after collection, urine was acidified to pH < 3 using 20% (vol/vol) H_2_SO_4_ to avoid the volatilization of N compounds, then stored at −20 °C. At the end of the experiment, urine samples were thawed at room temperature, pooled on an equal-volume basis by cows and periods, and stored at −20 °C for N analysis. The N concentration of urine was determined by the Kjeldahl procedure (method 4.1.1, [23]). For PD analysis (allantoin and uric acid), 500 mL of the pooled urine was filtered (MN 612 ¼, 320 mm Ø, Macherey-Nagel GmbH & Co. KG, Düren, Germany). From this filtrate, two aliquots of 20 mL were diluted (1:5, vol/vol) with double distilled water, and four aliquots (two aliquots each for determination of allantoin and uric acid) of 10 mL were taken and stored at −20 °C. The diluted aliquots (1:5, vol/vol) were further diluted with double distilled water to 1:25 for allantoin and 1:20 for uric acid analysis. The PD analysis was conducted using HPLC (JASCO HSS-1500, Groß-Umstadt, Germany) followed by peak area analysis with Galaxie Chromatography Software Version 1.10.0.5590 (Agilent Technologies).

### 2.3. Calculations and Statistical Analysis

The MCP synthesis was estimated based on the total PD excretion (MCPPD) according to Chen and Ørskov [24], using the following equation:

MCPPD (g/d) = PD absorption × 70/0.116 × 0.83 × 1000,
(1)

where PD absorption is in mmol/d, 70 is the N content of purines in mg/mmol, 0.116 is the ratio of purine-N to total-N in mixed rumen microbes, and 0.83 is the assumed digestibility of microbial purines. The PD absorption was calculated as follows:

PD absorption = (PD − 0.385 × BW^0.75^)/0.85, (2)

where PD is total excretion in g/d, and 0.385 × BW^0.75^ is the endogenous excretion of N, and BW is in kg. Additionally, MCP (g/d) was estimated from energy content of the diets (MCPME) and on digestible organic matter (MCPdOM) as follows:

MCPME (g/d) = DMI × 10.1 × ME,
(3)

MCPdOM (g/d) = OMI × ATTDOM × 156,
(4)

where DMI is in kg/d and is that reported by Schulz et al. [16], the ME is metabolizable energy content of the TMR in MJ/kg DM, estimated in vitro (RCS15 = 11.6, RCS30 = 11.4, RCS45 = 10.9, RCS60 = 10.7; [25]), 10.1 is the assumed value of MCP efficiency in g/MJ ME according to German protein evaluation system [26], OMI is organic matter (OM) intake in kg/d, ATTDOM is the apparent total tract digestibility of OM reported by Schulz et al. [16], and 156 is the assumed value of MCP efficiency in g/kg digestible OM according to GfE [26].

All statistical analyses were conducted using the software R (version 3.1.0, The R Foundation for Statistical Computing; Vienna, Austria). A summary of the data used for the correlations and the multivariate analysis is presented in Table 2.

The data evaluation started with the definition of an appropriate statistical mixed model, including the rations and periods as fixed factors. The animal group was regarded as a random factor. The data were assumed to be normally distributed and to be heteroscedastic due to the different levels of ration and period. These assumptions were based on graphical residual analysis. Also, the correlations of the measurement values due to the several levels of period were taken into account. Based on this model, a pseudo R^2^ value was calculated and an analysis of variances was conducted. Afterwards, a model selection (forward selection), based on Akaike information criterion values, was conducted to decide which covariates could be used for prediction. After that, covariates with a variance inflation factor of 10 or higher were removed in a stepwise fashion. Finally, non-significant covariates were omitted, based on an analysis of covariance. For the final model, a pseudo R^2^ value was calculated. For estimated values of MCP synthesis, multiple contrast tests were applied in order to compare the rations. After that, linear and quadratic contrasts were applied to check the shape of the dose–response curve. Significance was declared at *p* < 0.05, and tendencies with *p*-values between 0.05 and 0.10 were found.

## 3. Results

### 3.1. Estimated Microbial Crude Protein Synthesis

The effects of replacing MS with RCS on the estimated MCP are presented in Table 3. Increasing levels of RCS decreased the estimated MCPPD linearly (*p* < 0.001) from 1998 to 1707 g/d. Similarly, the estimated MCPME and MCPdOM reduced linearly (*p* < 0.001 for both) from 2729 to 2224 g/d and from 2328 to 2015 g/d, respectively.

### 3.2. Relationship Between OBCFA and Estimated Microbial Crude Protein Synthesis

The Pearson correlations of the proportions of individual or total OBCFA with the urinary parameters and estimated MCP synthesis are shown in Table 4. Only significant correlations (*p* < 0.05) are shown. The correlations are generally weak. The strongest negative correlation was found between the proportion of anteiso-C15:0 and the concentration of N in urine (*r* = −0.37). The strongest positive correlation was between the yield of iso-C15:0 and MCPdOM (*r* = 0.55). Most of the proportions of individual OBCFA, with the exception of iso-C14:0 and iso-C18:0, correlated positively with concentration of allantoin and total PD (*r* = 0.21 to 0.40). However, proportions of individual OBCFA correlated negatively with concentration of uric acid (r = −0.18 to −0.37), with the exception of iso-C18:0 with uric acid excretion (*r* = −0.22 to −0.34). Both iso-C14:0 and iso-C18:0 correlated negatively with concentrations of N, allantoin, and total PD in urine (*r* = −0.18 to −0.35). Surprisingly, the proportions of C17:0 and cis-9 C17:1 correlated negatively with MCPME and MCPdOM (*r* = −0.21 to −0.23). Moreover, all yields of individual OBCFA correlated positively with MCPME (*r* = 0.23 to 0.53) and MCPdOM (*r* = 0.21 to 0.55), whereas neither individual OBCFA nor total OBCFA correlated with MCPPD (Table 5). Finally, MCPME correlated with the yields of total BCFA, OCFA, and OBCFA (*r* = 0.47, 0.40, and 0.45, respectively). Similarly, MCPdOM correlated with the yields of total BCFA, OCFA, and OBCFA (*r* = 0.49, 0.42, and 0.48, respectively).

The multivariate models used to predict the concentration and daily excretion of urinary parameters, as well as the MCP from milk individual OBCFA proportions and yields, are summarized in Table 6. The proportion of iso-C17:0 was the main predictor of the concentrations of allantoin and total PD in urine (R^2^ = 0.67 and 0.64, respectively). The predictions of the concentrations of allantoin and total PD from OBCFA proportions were better than those based on yields. Furthermore, individual OBCFA had a better ability to predict daily allantoin and PD excretions than their concentrations. The prediction accuracy of MCPPD was high (R^2^ = 0.99) when the proportions of iso-C16:0 and cis-9 C17:1 were included, but no reliable equation was delivered based on the yields of individual OBCFA. The prediction accuracy of MCPME and MCPdOM (R^2^ = 0.24 and 0.21, respectively) from the proportions of cis-9 C17:1 and anteiso-C17:0 was low, whereas that of MCPME and MCPdOM was improved (R^2^ = 0.37 and 0.36, respectively) when the yields of iso-C15:0, cis-9 C17:1 and C18:0 were taken into account.

**Figure 1:**
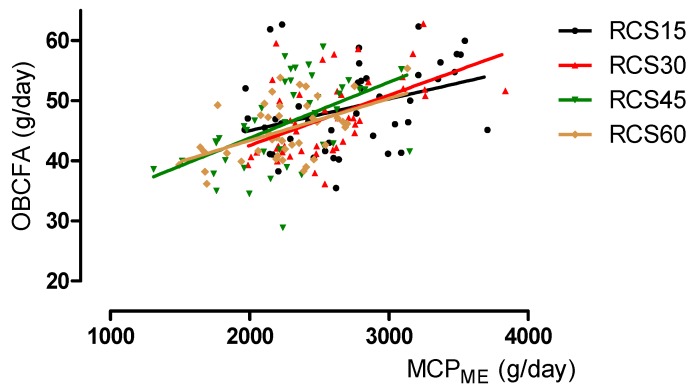
Relationship between the yield of odd- and branched-chain fatty acid (OBCFA) and microbial crude protein (MCP) synthesis, estimated based on intakes of metabolizable energy (MCPME), R^2^ = 0.27, *p* < 0.001. The total mixed rations (TMR) comprised forage and concentrates (0.75:0.25), with targeted ratios of red clover silage (RCS) in the TMR of 0.15 (RCS15), 0.30 (RCS30), 0.45 (RCS45), and 0.60 (RCS60) on a dry matter (DM) basis.

**Figure 2:**
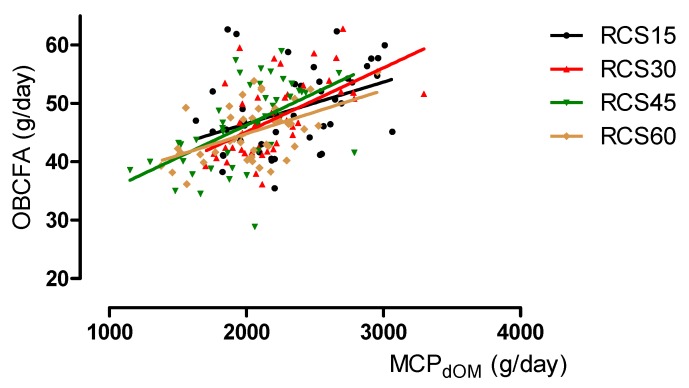
Relationship between the yield of odd- and branched-chain fatty acid (OBCFA) and microbial crude protein (MCP) synthesis, estimated based on intakes of digestible organic matter (MCPdOM), R^2^ = 0.29, *p* < 0.001. The total mixed rations (TMR) comprised forage and concentrates (0.75:0.25), with targeted ratios of red clover silage (RCS) in the TMR of 0.15 (RCS15), 0.30 (RCS30), 0.45 (RCS45), and 0.60 (RCS60) on a dry matter (DM) basis.

The linear regression equations used to estimate the urinary PD (concentration and total excretion) and MCP, based on the proportions and yields of total BCFA, OCFA, or OBCFA, are shown in Table 7. The PD concentration in urine was predicted moderately from the proportions of total BCFA, OCFA, or OBCFA (R^2^ = 0.57 to 0.64). The prediction reliabilities of the daily excretion of PD based on the proportions and yields of BCFA, OCFA, or OBCFA were low (R^2^ = 0.11 to 0.13). The prediction reliabilities of MCPPD based on the proportions and yields of BCFA, OCFA, or OBCFA were moderate (R^2^ = 0.50 to 0.56). However, the effects of BCFA, OCFA and OBCFA were not significant in the equations (*p* > 0.10). The accuracy of the predictions of MCPME and MCPdOM from proportions of BCFA, OCFA, or OBCFA were low (R^2^ = 0.12 to 0.16), whereas those based on yields of BCFA, OCFA, or OBCFA were improved (R^2^ = 0.22 to 0.34). Figure 1 shows the relationships of yield of OBCFA to MCPME for each TMR. This relationship produces the following regression equation: RCS15: y = 34.5 + 0.0053x (R^2^ = 0.13, *p* = 0.02); RCS30: y = 25.9 + 0.0084x (R^2^ = 0.25, *p* < 0.001); RCS45: y = 25.2 + 0.0093x (R^2^ = 0.26, *p* < 0.001); RCS60: y = 29.4 + 0.0070x (R^2^ = 0.25, *p* < 0.001). Figure 2 shows the relationships of the yield of OBCFA to MCPdOM. This relationship delivered the following regression equation: RCS15: y = 32.3 + 0.0071x (R^2^ = 0.16, *p* < 0.01); RCS30: y = 23.2 + 0.0110x (R^2^ = 0.30, *p* < 0.001); RCS45: y = 24.1 + 0.0111x (R^2^ = 0.29, *p* < 0.001); RCS60: y = 30.0 + 0.0074x (R^2^ = 0.25, *p* < 0.001). Both figures show a positive relationship between the estimated MCP synthesis and yield of OBCFA.

## 4. Discussion

### 4.1. Estimated Microbial Crude Protein Synthesis

Estimated MCPME synthesis was higher than MCPdOM and MCPPD was lowest. The requirement for the maintenance and milk production, according to GfE [26], was on average 3247 g/d utilizable CP (sum of MCP and ruminally undegraded feed CP). The proportion of estimated MCP synthesis in total utilizable CP averaged 55%, 75%, and 66% for MCPPD, MCPME, and MCPdOM, respectively. Thus, MCPPD seems to be an underestimate, whereas MCPME and MCPdOM are more realistic, because MCP normally supplies 60% to 85% of amino acids reaching the duodenum [1]. Although changes in the urinary excretion of PD closely reflect measurements of MCP flow to the duodenum, the total PD excretion and that of allantoin generally provides lower estimates of duodenal microbial N flow than direct measurements in the omasum or duodenum [3]. Though there were obvious differences in the calculated MCP between methods, the ranking of diet effects was not affected by the methods. The MCPME is greater than MCPdOM, likely due to the assumption of a constant efficiency of 10.1 g MCP/MJ ME, which does neither consider differences in the proportion of the disappearance of nutrients in the rumen and intestine, nor the reduction of digestibility and metabolizable energy at high feeding levels and passage rates in dairy cows. A decline of the ME content, with a 1.8% per unit increase in feeding level above ME requirement for maintenance, as assumed by AFRC [27], was not considered here. However, the calculation of MCPdOM was based on the real ATTDOM determined in the experimental cows [16], which explains why MCPdOM was lower than MCPME here. Therefore, it can be assumed that the MCPdOM values are the most realistic.

The estimated MCP flow to the duodenum, independent of the method used, reduced linearly by 13% to 19% with an increasing proportion of RCS in the diet. Considering that estimations of MCP in the present study were based primarily on the intake of DM and OM, differences in the calculated values of MCPME and MCPdOM between diets mostly reflect differences in the feed intake of cows. Moreover, the reduction of calculated MCPME synthesis also reflects the reduced ME content of the diets, whereas the reduction of MCPdOM synthesis reflects the slight reduction of OM content with increasing levels of RCS. Both the reduction of ME and OM were caused by the inclusion of RCS. The ATTDOM was similar among diets (71 ± 0.9%; [16]), and therefore is assumed to not have had an effect on the calculation of MCPdOM. The amount of synthesized MCP in the rumen depends on the availability of fermentable carbohydrates, N, and protein, and is limited by the available energy in the rumen for microbes. The reduction of the estimated MCP with increasing RCS in the diets can be explained by a decrease in the content of rapidly fermentable carbohydrates (sugar and starch), which reduced the availability of fermentable energy for microbes. Moreover, the reduced content of OM in the TMR with an increasing level of RCS resulted in reduced OM intake [16], and, in turn, may have reduced MCP synthesis. In addition, Schulz et al. [16] have reported a decreased milk protein concentration when increasing the proportion of RCS, which can be supported by the reduction in estimated MCP flow to the duodenum. Finally, in the in vitro study of Castro-Montoya et al. [28], with the same diets of this experiment, the concentration of purine bases and total N in liquid-associated microbes declined with an increasing RCS level, suggesting the negative impact of the feed on MCP synthesis. The latter agrees with the findings of the current estimation.

### 4.2. Relationship of OBCFA with Urinary Purine Derivates and Estimated Microbial Crude Protein Synthesis

The OBCFA are synthesized de novo by ruminal microbes and, therefore, are influenced by the rumen environment, microbial activity, and microbial community composition. It is, therefore, expected that differences in diet composition may elicit an effect on the rumen environment, and, therefore, the synthesis of MCP and OBCFA. Differences in estimated MCP yield and milk OBCFA composition were indeed achieved by feeding TMR containing different RCS and MS proportions. The coefficient of variation of proportions and yields of individuals OBCFA varied between 9.5% to 28.3% and between 16.1% to 28.8%, respectively. Additionally, as shown in the previous related study [18], a quadratic effect was found for BCFA in milk fat, with the lowest proportion of BCFA with diet RCS45 compared to diet RCS15 (1.78 vs. 1.82 g/100 g FA). Moreover, with the substitution of MS by RCS and SBM by wheat, in order to maintain similar concentrations of CP among the diets, the compositions of the diets differed to some extent. Therefore, increasing the RCS level caused a decrease in sugar (from 56.2 to 28.4 g/kg DM) and starch (from 232 to 170 g/kg DM) content, a slight increase in ADF concentration (from 212 to 244 g/kg DM), and a decrease in energy content [25]. Those changes in the diet would promote differences in the composition of microbial populations, and, expectedly, in OBCFA in milk. The present study was designed to assess the relationship of individual or total OBCFA in the milk with urinary variables and estimated rumen MCP synthesis. Because OBCFA are synthesized de novo by microorganisms of the rumen [6], and MCP synthesis is reflected by urinary PD excretion [3], a good relationship was expected between the urinary variables and OBCFA in the milk. However, the correlations obtained in this study show weak to moderate relationships of the proportions and yields of individual OBCFA in milk with the urine variables (N, uric acid, allantoin, and total PD). This is in agreement with results reported by Castro-Montoya et al. [12], who found relationships with *r* between −0.42 and 0.37. Yields of individual OBCFA and yields of total BCFA, OCFA, or OBCFA correlated positively (*r* = 0.21 to 0.55) with the estimated MCPME and MCPdOM. Both MCPME and MCPdMO respond basically to the same parameter, that is, energy intake. It is well known that higher energy intakes lead to an overall higher level of microbial protein synthesis, even though differences at the animal level might be difficult to identify. The fact that OBCFA correlates better to these two estimations of MCP might be an indication of the robustness of these FAs to predict changes in MCP, albeit at the expense of accuracy.

In this study, we additionally attempted to predict the excretion of PD and MCP synthesis based on multiple linear regressions using the proportions and yields of individual OBCFA. Similar to the study of Castro-Montoya et al. [12], FAs expressed as proportions in fat seemed to be better explanatory variables for PD-related variables than FAs expressed as yields. Furthermore, an interesting finding was that iso-C17:0 was related positively to PD excretion, while cis-9 C17:1 was related negatively to it, in agreement with the findings of Castro-Montoya et al. [12]. The fact that the same two FAs were identified as important parameters for PD excretion in both studies speaks about the potential relationships between these variables. This could serve as the basis for further research targeting those FAs.

The prediction of allantoin and PD concentration was better than the prediction of allantoin and PD daily excretion. It is not clear why this was the case, but it may be related to the additional error source provided by the quantification of the urine volume. The sole iso-C17:0 proportion delivered better equations for prediction and explained 64% to 67% of the variation in concentrations of allantoin and total PD, providing a more suitable predictor. Because allantoin accounted for 97% of the total PD concentration in urine [16], allantoin and PD were predicted with similar accuracy. Weak relationships (R^2^ = 0.10 to 0.14) of the concentrations of allantoin or total PD with proportions and yields of individual OBCFA in the milk were also found by Castro-Montoya et al. [12]. The MCPME and MCPdOM were only predicted moderately from yields of both iso-C15:0, cis-9 C17:1 and iso-C18:0, whereas, for MCPPD, no reliable equation could be established. However, MCPPD was best predicted from the proportions of both iso-C16:0 and cis-9 C17:1, in agreement with Castro-Montoya et al. [12].

The linear regression equations, based on the proportions of BCFA, OCFA or OBCFA, predicted the concentration of PD with the highest accuracy. On the contrary, predictions of the daily excretion of PD were low, while prediction equations for MCPPD based on concentrations and yields of BCFA, OCFA, or OBCFA were clearly better. It is unclear why the prediction of daily PD excretions appears to be so low, but it is possible that, as seen from the current results and previous studies, not all individual FA are robust/accurate predictors of MCP or its indicators. Therefore, when grouping the milk FAs, some antagonistic effects may occur, canceling out the predictive power of some FAs. The latter means that it could be that an individual FA reacts more sensitively than a group of FAs, because within a group, a compensating effect may occur, which means that an increase of one specific FA might be associated with the decrease of a similar FA. The improved prediction of the MCP from PD may reflect that OBCFA are better at predicting the overall process of microbial protein synthesis, rather than the excretion of specifically allantoin and uric acid. These phenomena deserve further attention in order to understand their mechanisms and better target individual or grouped OBCFA for the prediction of rumen fermentation parameters.

In general, the correlations and regressions demonstrate that the yields and concentrations of individual or total OBCFA are between low and moderate markers for MCP synthesis in the rumen. The results of this study are in agreement with Vlaeminck et al. [9], who found positive relationships of the flow of both microbial purine bases and diaminopimelic acid to the duodenum with milk yield of OBCFA. However, these are also contradictory observations, i.e., a negative correlation between the calculated MCP and OCFA was observed, with exception of iso-C15:0 and C15:0, as reported by Cabrita et al. [8]. However, it is difficult to identify whether this effect was confounded by other dietary components. Additionally, different proportions of OBCFA in the rumen and milk were also found [10]. It must be noted that PD excretions are only an indirect indicator for the duodenal flow of MCP, and that the relatively limited accuracy as indicator for MCP might contribute to the moderate relationship with the proportions and yields of individual and total OBCFA. Moreover, different dietary ratios of forage and concentrates [29] and differences in the diet composition of cows normally results in greater variation in the species composition of the microbial population in the rumen, which might explain the moderate relationships between OBCFA in the milk and estimated MCP synthesis in the rumen. However, as mentioned above, the substitution of MS by RCS resulted in significant differences in diet composition, which discards this aspect as a reason for the moderate relationships. Nevertheless, despite the fact that the correlation and regressions analyses included the data of animals within the same dietary treatment, relationships between OBCFA and estimated MCP remained between low and moderate, suggesting that factors other than differences in the composition of rumen microbial population might also be responsible for this disagreement [12]. In line with this, Fievez et al. [6] concluded that strong correlations between OBCFA and the duodenal flow of microbes might be valid when variation in the composition of microbial population is modest and independent of changes in composition of diets for cows.

### 4.3. Limitations of the Present Study

This chapter aims to briefly highlight important limitations of the present study related (1) to the use of PD for estimation of MCP flow to the duodenum, (2) the use of OBCFA as biomarker, and (3) the procedure used for the estimation of MCP synthesis.

The use of PD excreted in the urine has been suggested as a reliable method to estimate microbial flow to the duodenum [2,3,4,5], and is a good alternative to avoid some of the difficulties associated to animal welfare concerns [30], in vivo measurements using cannulated animals for duodenal or omasal sampling [31], the variability in digesta sampling and flow, markers for digesta flow, and microbes or considerations on the ratios of marker to N concentrations in rumen microbes [30]. However, there are a number of errors due to feed purines that escape rumen degradation, variations in the content of rumen microbes, the variable partitioning of PD between renal, mammary, and enteric excretory routes, and the excretion of PD due to endogenous purine metabolism [30]. Additionally, the assumption of a constant purine-N to total-N ratio in mixed microbes has been topic of criticism, because ratios are subject to considerable variation [32]. In the present study, a standard ratio of 0.116 [24] was used. However, some studies have demonstrated that this ratio for ruminal microbes markedly varies depending on the microbial composition [33] and type of diets [31]. The latter indicates that the estimated MCP synthesis based on PD could be biased by the use of the mentioned standard ratio, therefore, results based on PD must be interpreted with caution and used to assess relative differences, rather than quantitative estimates of microbial protein supply.

Some studies have studied and suggested the use of OBCFA in milk [6,8,9,10,11,12] and in the rumen [34] as biomarkers for microbial synthesis in the rumen. OBCFA are synthesized de novo by ruminal bacteria and incorporated in their cell membrane, suggesting a direct relation with bacterial biomass [6]. Although, the contribution of OBCFA endogenous synthesis in milk from dairy cows is assumed to be negligible [35,36], OCFA and their anteiso isomers can be synthesized in the mammary gland through the incorporation of propionyl-CoA instead of acetyl-CoA, or methylmalonyl-CoA instead of malonuyl-CoA, respectively [9]. Milk secretion of OCFA was found to be higher than that of these FA in duodenal flow [37]. Similarly, Dewhurst et al. [11] found a greater yield of C15:0, C17:0, and iso-C17:0 in milk than in duodenal flow, while Liu et al. [10] reported higher contents of OCFA (except those of C15:0) in the milk than in the rumen, and Vlaeminck et al. [38] detected that the OBCFA profile in milk was enriched in C15:0, iso-C17:0, anteiso-C17:0, and cis-9 C17:1, as compared with duodenal flow. All these observations suggest the occurrence of post-ruminal synthesis or modification, such as the de novo synthesis, desaturation, and elongation [38] of certain OBCFA in animal tissue. Moreover, the lactation stage is a factor that could have affected the results of OBCFA in milk. The mobilization of adipose tissue during a negative energy balance can contribute to milk OBCFA. Additionally, mobilization can be also highly relevant if the body fat has a significantly different OBCFA content than the milk fat. However, the cows were studied after the peak of lactation and gaining weight [18]. Moreover, the incorporation of OBCFA in body tissue due to the daily weight gain of cows could have also contributed to the weak relationships between milk OBCFA and the urinary parameters. However, this could be hardly relevant, because the amount of fat deposition can be considered greatly lower compared to the milk fat yield of the cows (1.32 kg/d, [16]). All the mentioned factors could dilute or enrich specific OBCFA in milk when their absorption is not different and could have caused the weak to moderate relationship of individual and total OBCFA with urinary parameters and estimated MCP synthesis. Although the post-ruminal synthesis and modification of the OBCFA profile might hamper the use of OBCFA as a tool for the estimation of ruminal MCP synthesis, this effect is generally very small in absolute terms in milk as compared with duodenal content [38], and, consequently, the OBCFA profile in milk still provides information on rumen function and MCP synthesis.

The MCP synthesis in this study was estimated based on published equations [24,26] and not on the measured values of MCP. However, the equations used for MCP synthesis have been derived from multiple studies with different animals and dietary conditions, which is more robust for prediction compared with an equation derived from a single study [31]. The best and most reliable determination of MCP synthesis in the rumen should be based on measurements of the intestinal flow in duodenal-cannulated cows. However, possible underestimations of duodenal FA flows by methodological limitations may have occurred when recovery values greater than 1 were observed [38]. Even when using duodenal-cannulated cows, Vlaeminck et al. [38] reported that the profile of milk OBCFA differed from the profile in duodenal content. In general, the values presented for the effect of increasing RCS in the diets on MCP synthesis should not be taken as those identical with those in vivo, but rather as relative values to evaluate the protein providing potential of diets. Even though these results should be interpreted with care, and that equations are not recommended to be directly used to estimate MCP synthesis based on OBCFA, they give an indication about the effect of including RCS in the diets of dairy cows. Finally, the effect of replacing MS with RCS on the estimated MCP synthesis of this study matches with the reduction in concentration and yield of milk protein [16], the estimated flow of utilizable CP at the duodenum, based on an in vitro technique [25] or that calculated with an official equation of the German protein evaluation system [39], and the concentration of purine bases and total N in liquid-associated microbes in vitro [28]. The latter indicates the relative reliability of the approaches used to estimate MCP synthesis.

In general, the use of PD for the estimation of microbial flow, which is still subject to some unsafe assumptions, the uncertainty of the OBCFA origin, and its moderate relation to MCP, and the estimation of MCP based on published equations, and not on direct measurements in vivo, are factors that limit strong and final conclusions in this study. However, it can be argued that while these limitations mask the conclusions, true relations between the parameters studied may be inferred. Therefore, the limits, as well as the potential of the procedures, have been evaluated and presented here.

## 5. Conclusions

The inclusion of RCS in diets for dairy cows increases the proportions of OBCFA in milk fat. However, RCS reduces the amount of protein provided to cows via the reduction of MCP flow to the duodenum. MCP synthesis and urinary PD excretion can be only moderately predicted by yields and concentrations of individual or total OBCFA in cow’s milk, likely caused by the variable transfer of microbial OBCFA to milk and changes in the composition of the microbial community. Nevertheless, there is evidence that milk OBCFA can be robust predictors of changes in MCP synthesis and flow to the duodenum in dairy cows. Therefore, milk OBCFA profiles continue to be a promising, non-invasive method. However, more research is needed to better describe the transfer of OBCFA from the rumen to the milk to improve the accuracy of prediction.

## Figures and Tables

**Table 1:** Ingredients and chemical composition of the total mixed rations (TMR).

Item	TMR ^1^			
	RCS15	RCS30	RCS45	RCS60
Ingredients (g/kg DM)				
Maize silage	610	466	316	162
Red clover silage	136	275	421	571
Soybean meal	159	108	55.0	-
Wheat	-	54.9	110	168
Lupine seed	85.9	87.0	88.8	89.6
Premix ^2^	9.1	9.1	9.2	9.4
Chemical composition (g/kg DM)				
Crude ash	60.5	70.7	84.2	93.2
Organic matter ^3^	940	929	916	907
Crude protein	172	174	173	175
Ether extract	21.4	21.3	20.5	20.2
Sugar	56.2	29.3	25.1	28.4
Starch	232	205	180	170
Neutral detergent fibre (aNDFom) ^4^	340	332	342	341
Acid detergent fibre (ADFom) ^5^	212	219	239	244
Lignin (sa)	28.0	32.4	36.9	41.0

^1^ The TMR was comprised of forage and concentrates (0.75:0.25), with targeted ratios of red clover silage (RCS) in TMR of 0.15 (RCS15), 0.30 (RCS30), 0.45 (RCS45), and 0.60 (RCS60) on a dry matter (DM) basis.^2^ Premix contained salt, minerals, and vitamins. ^3^ Organic matter calculated as 1000–CA, where CA is crude ash and is in g/kg DM. ^4^ aNDFom was assayed using a heat-stable amylase and expressed exclusive of residual ash. ^5^ ADFom was expressed exclusive of residual ash.

**Table 2:** Summary statistics of variables utilized for the multivariate analysis (n = 176).

Item	Min.	Max.	Mean	SD 1	CV 2 (%)
OBCFA 3 proportions (g/100g fatty acid)					
Iso-C14:0	0.050	0.130	0.084	0.016	19.4
Iso-C15:0	0.140	0.270	0.221	0.022	10.0
Anteiso-C15:0	0.260	0.540	0.437	0.041	9.46
C15:0	0.730	1.330	1.033	0.115	11.1
Iso-C16:0	0.140	0.320	0.226	0.036	16.1
Iso-C17:0	0.220	0.370	0.295	0.031	10.4
Anteiso-C17:0	0.340	0.560	0.437	0.046	10.6
C17:0	0.440	0.750	0.564	0.066	11.7
cis-9 C17:1	0.140	0.410	0.216	0.049	22.7
Iso-C18:0	0.010	0.110	0.058	0.016	28.3
OBCFA yields (g/d)					
Iso-C14:0	0.604	1.783	1.098	0.254	23.1
Iso-C15:0	1.358	4.412	2.895	0.526	18.2
Anteiso-C15:0	2.414	9.017	5.736	1.027	17.9
C15:0	5.884	19.09	13.51	2.252	16.7
Iso-C16:0	1.559	5.091	2.966	0.659	22.2
Iso-C17:0	1.710	5.770	3.862	0.685	17.7
Anteiso-C17:0	2.213	9.711	5.739	1.081	18.8
C17:0	3.169	10.63	7.364	1.185	16.1
cis-9 C17:1	1.157	6.050	2.815	0.686	24.4
Iso-C18:0	0.119	1.650	0.755	0.217	28.8
Urine parameters					
Nitrogen (g/L)	5.093	13.43	8.978	1.593	17.7
Uric acid (mmol/L)	0.313	0.804	0.458	0.80	17.4
Allantoin (mmol/L)	7.450	19.30	13.75	2.389	17.4
Total purine derivates (mmol/L)	8.010	19.70	14.21	2.380	16.7
Nitrogen (g/d)	61.70	424.7	220.3	64.00	29.1
Uric acid (mmol/d)	3.039	24.31	11.45	3.967	34.7
Allantoin (mmol/d)	102.3	677.7	337.8	99.22	29.4
Total purine derivates (mmol/d)	105.4	699.3	349.2	101.8	29.2
MCP 4 synthesis (g/d)					
MCPPD	1205	3448	1812	459	25.3
MCPME	1309	3837	2450	456	18.6
MCPdOM	1151	3296	2137	372	17.4

^1^ SD, Standard deviation. ^2^ CV, coefficient of variation. ^3^ OBCFA, odd- and branched-chain fatty acid. ^4^ MCP, Microbial crude protein, estimated based on urinary excretion of purine derivates (MCPPD), and intakes of metabolizable energy (MCPME) or intakes of digestible organic matter (MCPdOM).

**Table 3:** Effect of feeding total mixed rations (TMR) on urinary purine derivate (PD) excretion and estimated microbial crude protein (MCP) synthesis (mean ± SD).

MCP ^1^ Synthesis (g/d)	TMR ^2^				*p*-Value ^3^	
	RCS15	RCS30	RCS45	RCS60	Linear	Quadratic
MCPPD	1998 ^a^ ± 437	1840 ^b^ ± 544	1541 ^c^ ± 319	1707 ^b,c^ ± 381	<0.001	<0.001
MCPME	2729 ^a^ ± 486	2580 ^b^ ± 393	2266 ^c^ ± 392	2224 ^c^ ± 342	<0.001	0.01
MCPdOM	2328 ^a^ ± 397	2208 ^b^ ± 324	1999 ^c^ ± 345	2015 ^c^ ± 324	<0.001	0.007

^a,b,c^ Different superscript letters differ among TMR types (*p* < 0.05). ^1^ Estimated MCP, based on the urinary excretion of PD (MCPPD) and intakes of metabolizable energy (MCPME) or intakes of digestible organic matter (MCPdOM). ^2^ The TMR comprised of forage and concentrates (0.75:0.25), with targeted ratios of red clover silage (RCS) in the TMR of 0.15 (RCS15), 0.30 (RCS30), 0.45 (RCS45), and 0.60 (RCS60) on a dry matter (DM) basis. ^3^ Probability of a linear or quadratic effect of incrementing the proportion of RCS in the TMR.

**Table 4:** Correlation ^1^ coefficients between proportions of individual fatty acids (FA, g/100 g FA) and the urine parameters and estimated microbial crude protein synthesis (MCP, g/d).

OBCFA ^2^ Proportions									MCP Synthesis ^4^		
	Nitrogen (g/L)	Uric Acid (mmol/L)	Allantoin (mmol/L)	Total PD ^3^ (mmol/L)	Nitrogen (g/d)	Uric Acid (mmol/d)	Allantoin (mmol/d)	Total PD (mmol/d)	MCPPD	MCPME	MCPdOM
Iso-C14:0	−0.18		−0.21	−0.21							
Iso-C15:0	0.23	−0.26	0.22	0.21	0.19						
Anteiso-C15:0	0.40	−0.37	0.36	0.35		−0.30					
C15:0		−0.18	0.26	0.25			0.18				
Iso-C16:0											
Iso-C17:0	0.28	−0.29	0.36	0.35		−0.23					
Anteiso-C17:0	0.44	−0.26	0.39	0.39		−0.31					
C17:0										−0.21	
cis-9 C17:1	−0.34				−0.20					−0.23	−0.22
Iso-C18:0	−0.35		−0.29	−0.29		0.19					
Iso-C15:0 + iso-C17:0	0.31	−0.33	0.35	0.34		−0.22					
C17:0 + cis-9 C17:1					−0.19					−0.27	−0.25
Total iso-BCFA ^5^											
Total anteiso-BCFA	0.41	−0.31	0.42	0.41		−0.31					
Total BCFA	0.27	−0.32	0.27	0.25		−0.25					
Total OCFA ^6^											
Total OBCFA		−0.29	0.22	0.21		−0.23					

^1^ Only significant correlations with *p* < 0.05 are presented. ^2^ OBCFA, odd- and branched-chain FAs. ^3^ PD, purine derivates as the sum of uric acid and allantoin. ^4^ MCP estimation based on the urinary excretion of PD (MCPPD) and intakes of metabolizable energy (MCPME) or intakes of digestible organic matter (MCPdOM). ^5^ BCFA, branched-chain FAs as the sum of iso-C14:0, iso-C15:0, iso-C16:0, iso-C17:0, iso-C18:0, anteiso-C15:0 and anteiso-C17:0. ^6^ OCFA, odd-chain FAs as the sum of C15:0, C17:0 and cis-9 C17:1.

**Table 5:** Correlation ^1^ coefficients between yields of individual fatty acids (FA, g/d) and the urine parameters and estimated microbial crude protein synthesis (MCP, g/d).

OBCFA ^2^ Yields									MCP ^4^ Synthesis		
	Nitrogen (g/L)	Uric Acid (mmol/L)	Allantoin (mmol/L)	Total PD ^3^ (mmol/L)	Nitrogen (g/d)	Uric Acid (mmol/d)	Allantoin (mmol/d)	Total PD (mmol/d)	MCPPD	MCPME	MCPdOM
Iso-C14:0		0.19				0.18				0.31	0.32
Iso-C15:0	0.21		0.18	0.18	0.20		0.20	0.19		0.53	0.55
Anteiso-C15:0	0.31		0.25	0.25						0.49	0.52
C15:0										0.42	0.45
Iso-C16:0		0.19								0.29	0.29
Iso-C17:0	0.23		0.25	0.25						0.40	0.43
Anteiso-C17:0	0.31		0.25	0.26						0.41	0.44
C17:0										0.35	0.36
cis-9 C17:1	−0.23	0.20								0.23	0.24
Iso-C18:0	−0.28	0.25	−0.24	−0.23		0.26				0.23	0.21
Iso-C15:0 + iso-C17:0	0.23		0.23	0.24						0.48	0.51
C17:0 + cis-9 C17:1										0.32	0.33
Total iso-BCFA ^5^						0.21				0.43	0.44
Total anteiso-BCFA	0.26		0.23	0.23						0.46	0.48
Total BCFA										0.47	0.49
Total OCFA ^6^										0.40	0.42
Total OBCFA										0.45	0.48

^1^ Only significant correlations with *p* < 0.05 are presented. ^2^ OBCFA, odd- and branched-chain FAs. ^3^ PD, purine derivates as the sum of uric acid and allantoin. ^4^ MCP estimation based on the urinary excretion of PD (MCPPD) and intakes of metabolizable energy (MCPME) or intakes of digestible organic matter (MCPdOM). ^5^ BCFA, branched-chain FAs as the sum of iso-C14:0, iso-C15:0, iso-C16:0, iso-C17:0, iso-C18:0, anteiso-C15:0 and anteiso-C17:0. ^6^ OCFA, odd-chain FAs as the sum of C15:0, C17:0 and cis-9 C17:1.

**Table 6:** Parameter estimates of regression equations between individual milk odd- and branched-chain fatty acid (1) proportions (g/100 g fatty acid), and (2) yields (g/d) to predict urine parameters and estimated microbial crude protein (MCP ^1^) synthesis.

Item	Equation		R^2^
Allantoin (mmol/L)	(1)	6.15 *** + 25.5 × iso-C17:0 ***	0.67
	(2)	11.1 *** + 1.98 × iso-C17:0 *** − 1.36 × iso-C15:0 ** − 1.45 × iso-C18:0 *	0.49
Total PD ^2^ (mmol/L)	(1)	6.81 *** + 24.8 × iso-C17:0 ***	0.64
	(2)	11.5 *** + 1.98 × iso-C17:0 *** − 1.33 × iso-C15:0 ** − 1.53 × iso-C18:0 **	0.47
Allantoin (mmol/d)	(1)	524 *** − 643 × anteiso-C17:0 *** − 554 × cis-9 C17:1 *** + 695 × iso-C17:0 *	0.16
	(2)	189 ** + 146 × iso-C15:0 *** − 32.1 × anteiso-C17:0 *** − 33.3 × iso-C16:0 *	0.22
Total PD (mmol/d)	(1)	552 *** − 676 × anteiso-C17:0 *** − 567 × cis-9 C17:1 *** + 693 × iso-C17:0 *	0.15
	(2)	197 ** + 151 × iso-C15:0 *** − 33.8 × anteiso-C17:0 *** − 33.5 × iso-C16:0 *	0.21
MCPPD (g/d)	(1)	1308 *** − 2525 × iso-C16:0 *** + 4973 × cis-9 C17:1 ***	0.99
	(2)	No delivered equation	−
MCPME (g/d)	(1)	3890 *** − 3172 × cis-9 C17:1 *** − 1824 × anteiso-C17 ***	0.24
	(2)	1154 *** + 480 × iso-C15:0 *** − 123 × cis-9 C17:1 * + 310 × iso-C18:0 *	0.37
MCPdOM (g/d)	(1)	3358 *** − 2896 × cis-9 C17:1 *** − 1411 × anteiso-C17:0 **	0.21
	(2)	1008 *** + 424 × iso-C15:0 *** − 110 × cis-9 C17:1 * + 269 × iso-C18:0 *	0.36

^1^ MCP estimated based on urinary excretion of purine derivates (MCPPD), and intakes of metabolizable energy (MCPME) or intakes of digestible organic matter (MCPdOM). ^2^ PD, purine derivates as the sum of uric acid and allantoin. * *p* < 0.05, ** *p* < 0.01, and *** *p* < 0.001 indicate the significance of intercepts or individual FAs.

**Table 7:** Parameter estimates of the regression equations of grouped odd- and branched-chain fatty acid (1) proportions (g/100 g fatty acid), and (2) yields (g/d) with the excretion of purine derivates (PD ^1^) and estimated microbial protein synthesis (MCP ^2^).

Item	Equation		R^2^
Total PD (mmol/L)	(1)	8.76 *** + 3.02 × BCFA *	0.56
	(1)	10.5 *** + 1.94 × OCFA ^†^	0.67
	(1)	6.70 ** + 2.06 × OBCFA **	0.65
	(2)	12.3 *** + 0.07 × BCFA ^†^	0.55
	(2)	12.6 *** + 0.06 × OCFA	0.60
	(2)	12.3 *** + 0.04 × OBCFA	0.57
Total PD (mmol/d)	(1)	409 *** − 40.7 × BCFA	0.12
	(1)	276 * + 33.8 × OCFA	0.11
	(1)	342 * – 1.23 × OBCFA	0.11
	(2)	248 *** + 3.99 × BCFA ^†^	0.13
	(2)	244 *** + 3.97 × OCFA *	0.13
	(2)	240 *** + 2.13 × OBCFA *	0.13
MCPPD (g/d)	(1)	1620 ** + 83.7 × BCFA	0.49
	(1)	2027 *** − 141 × OCFA	0.51
	(1)	1824 ** – 14.9 × OBCFA	0.52
	(2)	1487 *** + 12.4 × BCFA	0.52
	(2)	1534 *** + 10.1 × OCFA	0.56
	(2)	1496 *** + 5.94 × OBCFA	0.54
MCPME (g/d)	(1)	3265 *** − 493 × BCFA *	0.15
	(1)	3240 *** − 460 × OCFA ^†^	0.16
	(1)	3640 *** − 346 × OBCFA *	0.16
	(2)	1140 *** + 56.0 × BCFA ***	0.32
	(2)	1423 *** + 41.6 × OCFA ***	0.22
	(2)	1199 *** + 26.1 × OBCFA ***	0.27
MCPdOM (g/d)	(1)	2730 *** − 355 × BCFA	0.12
	(1)	2746 *** − 351 × OCFA	0.13
	(1)	3030 *** − 258 × OBCFA ^†^	0.13
	(2)	962 *** + 50.5 × BCFA ***	0.34
	(2)	1207 *** + 38.4 × OCFA ***	0.22
	(2)	1009 *** + 23.8 × OBCFA ***	0.29

^1^ PD, purine derivates as the sum of uric acid and allantoin. ^2^ Estimated MCP, based on the urinary excretion of PD (MCPPD) and intakes of metabolizable energy (MCPME) or intakes of digestible organic matter (MCPdOM). ^†^ 0.05 > *p* < 0.10, * *p* < 0.05, ** *p* < 0.01 and *** *p* < 0.001 indicate the significance of intercepts or groups of fatty acids (FA), otherwise they are not significant, i.e., *p* > 0.10. BCFA, branched-chain FAs as the sum of iso-C14:0, iso-C15:0, iso-C16:0, iso-C17:0, iso-C18:0, anteiso-C15:0 and anteiso-C17:0. OCFA, odd-chain FAs as the sum of C15:0, C17:0 and cis-9 C17:1. OBCFA, odd- and branched-chain FAs.

## References

[1] Storm E., Ørskov E.R., Smart R. (1983). The nutritive value of rumen micro-organism in ruminants. 2. The apparent digestibility and net utilization of microbial N for growing lambs. Br. J. Nutr..

[2] Südekum K.-H., Brüsemeister F., Schröder A., Stangassinger M. (2006). Effects of amount of intake and stage of forage maturity on urinary allantoin excretion and estimated microbial crude protein synthesis in the rumen of steers. J. Anim. Physiol. Anim. Nutr..

[3] Tas B.M., Susenbeth A. (2007). Urinary purine derivates excretion as an indicator of in vivo microbial N flow in cattle: A review. Livest. Sci..

[4] Ahnert S., Dickhoefer U., Schulz F., Susenbeth A. (2015). Influence of ruminal quebracho tannin extract infusion on apparent nutrient digestibility, nitrogen balance, and urinary purine derivatives excretion in heifers. Livest. Sci..

[5] Henke A., Dickhoefer U., Westreicher-Kristen E., Knappstein K., Molkentin J., Hasler M., Susenbeth A. (2017). Effects of quebracho tannin extract on feed intake, digestibility, excretion of urinary purine derivatives and milk production in lactating dairy cows. Arch. Anim. Nutr..

[6] Fievez V., Colman E., Castro-Montoya J.M., Stefanov I., Vlaeminck B. (2012). Milk odd- and branched-chain fatty acids as biomarkers of rumen function-An update. Anim. Feed Sci. Technol..

[7] Dewhurst R.J., Davis D.R., Merry R.J. (2000). Microbial protein supply from the rumen. Anim. Feed Sci. Technol..

[8] Cabrita A.R.J., Fonseca A.J.M., Dewhurst R.J., Gomes E. (2003). Nitrogen supplementation of corn silages. 2. Assessing rumen function using fatty acid profiles of bovine milk. J. Dairy Sci..

[9] Vlaeminck B., Dufour C., Van Vuuren A.M., Cabrita A.R.J., Dewhurst R.J., Demeyer D., Fievez V. (2005). Use of odd and branched-chain fatty acids in rumen contents and milk as a potential microbial marker. J. Dairy Sci..

[10] Liu K., Li Y., Xin H., Zhang Y., Li G. (2019). Relations of ruminal fermentation parameters and microbial matters to odd- and branched-chain fatty acids in rumen fluid of dairy cows at different milk stages. Animals..

[11] Dewhurst R.J., Moorby J.M., Vlaeminck B., Fievez V. (2007). Apparent recovery of duodenal odd- and branched faty acids in milk dairy cows. J. Dairy Sci..

[12] Castro-Montoya J., Henke A., Molkentin J., Knappstein K., Susenbeth A., Dickhoefer U. (2016). Relationship between milk odd and branched-chain fatty acids and urinary purine derivatives in dairy cows supplemented with quebracho tannins—A study to test milk fatty acids as predictors of rumen microbial protein synthesis. Anim. Feed Sci. Technol..

[13] Broderick G.A., Walgenbach R.P., Maignan S. (2001). Production of lactating dairy cows fed alfalfa or red clover silage at equal dry matter or crude protein contents in the diet. J. Dairy Sci..

[14] Dewhurst R.J., Davies L.J., Kim E.J. (2010). Effects of mixtures of red clover and maize silages on the partitioning of dietary nitrogen between milk and urine by dairy cows. Animal.

[15] Moorby J.M., Ellis N.M., Davies D.R. (2016). Assessment of dietary ratios of red clover and corn silages on milk production and milk quality in dairy cows. J. Dairy Sci..

[16] Schulz F., Westreicher-Kristen E., Knappstein K., Molkentin J., Susenbeth A. (2018). Replacing maize silage plus soybean meal with red clover silage plus wheat in diets for lactating dairy cows. J. Dairy Sci..

[17] Federal Republic of Germany (2014). Tierschutzgesetz. https://www.gesetze-im-internet.de/tierschg/BJNR012770972.html.

[18] Schulz F., Westreicher-Kristen E., Molkentin J., Knappstein K., Susenbeth A. (2018). Effect of replacing maize silage with red clover silage in the diet on milk fatty acid composition in cows. J. Dairy Sci..

[19] IDF (International Dairy Federation) (2010). Milk—Determination of Fat Content—Gravimetric Method (Reference Method). Standard 1 (ISO 1211).

[20] IDF (International Dairy Federation) (2002). Milk Fat—Preparation of Fatty Acid Methyl Esters. Standard 182 (ISO 15884).

[21] IDF (International Dairy Federation) (2002). Milk Fat—Determination of the Fatty Acid Composition by Gas-Liquid Chromatography. Standard 184 (ISO 15885).

[22] Molkentin J., Giesemann A. (2007). Differentiation of originally and conventionally produced milk by stable isotope and fatty acid analysis. Anal. Bioanal. Chem..

[23] VDLUFA (Verband Deutscher Landwirtschaftlicher Untersuchungs- und Forschungsanstalten) (2007). Handbuch der Landwirtschaftlichen Versuchs- und Untersuchungsmethodik (VDLUFA Methodenhandbuch), Bd. III. Die chemische Untersuchung von Futtermitteln.

[24] Chen X.B., Ørskov E.R. (2003). Research on Urinary Excretion of Purine Derivates in Ruminants: Past, present and Future.

[25] Westreicher-Kristen E., Blank R., Schulz F., Susenbeth A. (2017). Replacing maize silage with red clover silage in total mixed rations for dairy cows: In vitro ruminal fermentation characteristics and associative effects. Anim. Feed Sci. Technol..

[26] GfE (Gesellschaft für Ernährungsphysiologie) (2001). Empfehlungen zur Energie- und Nährstoffversorgung der Milchkühe und Aufzuchtrinder.

[27] AFRC (Agricultural and Food Research Council) (1993). Energy and protein requirements of ruminants. An Advisory Manual Prepared by the AFRC Technical Committee on Response to Nutrients.

[28] Castro-Montoya J., Witzig M., Rahman M., Westreicher-Kristen E., Dickhoefer U. (2018). Replacing maize silage with red clover silage in total mixed rations of dairy cows: Ruminal fermentation, microbial protein synthesis and composition of microbial community in vitro. J. Anim. Physiol. Anim. Nutr..

[29] Zhang Y., Liu K., Hao X., Xin H. (2017). The relationships between odd- and branched-chain fatty acids to ruminal fermentation parameters and bacterial populations with different dietary ratios of forage and concentrate. J. Anim. Physiol. Anim. Nutr..

[30] Shingfield K.J. (2000). Estimationof microbial protein supply in ruminant animals based on renal and mammary purine metabolite excretion. A review. J. Anim. Feed Sci..

[31] Firkins J.L., Hristov A.N., Hall M.B., Varga G.A., St-Pierre N.R. (2006). Integration of ruminal metabolism in dairy cattle. J. Dairy Sci..

[32] Clark J.H., Klusmeyer T.H., Cameron M.R. (1992). Microbial protein synthesis and flows of nitrogen fractions and amino acid nutrition in dairy cattle. J. Dairy Sci..

[33] Dickhoefer U., Ahnert S., Susenbeth A. (2016). Effects of quebracho tannin extract on rumen fermentation and yield and composition of microbial mass in heifers. J. Anim. Sci..

[34] Liu K., Hao X., Li Y., Luo G., Zhang Y., Xin H. (2017). The relantionship between odd- and branched-chain fatty acids and microbial nucleic acid bases in rumen. Asian-Australas J. Anim. Sci..

[35] Croom W.J., Bauman D.E., Davis C.L. (1981). Methylmalonic acid in low-fat milk syndrome. J. Dairy Sci..

[36] Massart-Leën A.M., Roets E., Peeters G., Verbeke R. (1983). Propionate for fatty acid synthesis by the mammary gland of the lactating goat. J. Dairy Sci..

[37] Vlaeminck B., Fievez V., Cabrita A.R.J., Fonseca A.J.M., Dewhurst R.J. (2006). Factors affecting odd- and branched-chain fatty acids in milk: A review. Anim. Feed Sci. Technol..

[38] Vlaeminck B., Gervais R., Rahman M.M., Gadeyne F., Gorniak M., Doreau M., Fievez V. (2015). Postruminal synthesis modifies the odd- and branched-chain fatty acid profile from the duodenum to milk. J. Dairy Sci..

[39] Westreicher-Kristen E., Südekum K.-H. (2018). Replacing maize silage plus soybean meal with red clover silage plus wheat grain in diets of dairy cows: Modelling the utilizable crude protein at the duodenum, a precursor to metabolizable protein. Anim. Feed Sci. Technol..

